# Real-world comparison of GLP-1 agonists versus physical activity in metabolic dysfunction-associated steatotic liver disease

**DOI:** 10.1186/s12876-026-04626-7

**Published:** 2026-02-25

**Authors:** Jason N. Chen, Bulent Tolga Delibasi, James Wang, Thomas Tran, Connie Hu, Charles W. Randall

**Affiliations:** 1 Department of Internal Medicine, The University of Texas Health Science Center at San Antonio, San Antonio, TX USA; 2 School of Medicine, The University of Texas Health Science Center at San Antonio, San Antonio, TX USA; 3 Department of Internal Medicine, The University of Texas at Tyler, Tyler, TX USA; 4 Gastroenterology Clinic of San Antonio, San Antonio, TX USA

**Keywords:** Metabolic syndrome, Lifestyle modification, Weight loss, Liver disease, Liver fibrosis

## Abstract

**Background:**

Glucagon-like peptide-1 (GLP-1) receptor agonists have metabolic and hepatic benefits in metabolic dysfunction–associated steatotic liver disease (MASLD), but their additive benefit compared to lifestyle modification only in real-world settings remains uncertain. This study compared the effects of GLP-1 agonist therapy and physical activity on body mass index (BMI) and noninvasive liver metrics in a predominantly Hispanic clinical population with MASLD.

**Methods:**

This is a retrospective longitudinal study of 202 adults with MASLD evaluated at an outpatient gastroenterology clinic. BMI, controlled attenuation parameter (CAP), liver stiffness (kPa), and Fibrosis-4 (FIB-4) indices were analyzed at baseline and follow-up (1–2 years). Primary analyses compared follow up outcomes between GLP-1 therapy and an unmonitored physical activity comparator group; exploratory analyses assessed whether effects differed by sex or ethnicity.

**Results:**

Among 193 patients with complete BMI data and 131 with liver metrics, GLP-1 agonist therapy was associated with a greater reduction in BMI (–1.47 kg/m²; 95% CI, − 2.54 to − 0.41; *p* = 0.007) and in FIB-4 indices (–0.29; 95% CI, − 0.56 to − 0.03; *p* = 0.029) compared with physical activity only. Adjusted differences in CAP (–3.31 dB/m) and liver stiffness (+ 0.01 kPa) were not statistically significant.

**Conclusion:**

In this real-world MASLD cohort, GLP-1 therapy was associated with greater baseline-adjusted improvements in BMI and FIB-4 compared with physical activity alone, while CAP and liver stiffness were not statistically significant. These findings provide supportive real-world evidence for GLP-1 associated metabolic benefit and warrant confirmation of hepatic effects in larger prospective studies, including cohorts with substantial Hispanic representation.

**Supplementary Information:**

The online version contains supplementary material available at 10.1186/s12876-026-04626-7.

## Introduction

Metabolic syndrome, a cluster of conditions including central obesity, insulin resistance, dyslipidemia, and hypertension, is a growing global health concern. In the United States, the prevalence of obesity continues to rise, with more than 40% of adults currently affected [1]. Obesity plays a central role in the development of metabolic dysfunction and is strongly associated with increased cardiovascular risk, type 2 diabetes mellitus, and chronic liver disease [2].

One major hepatic manifestation of metabolic syndrome is Metabolic Dysfunction-Associated Steatotic Liver Disease (MASLD). MASLD is now the most common cause of chronic liver disease worldwide, with prevalence estimates of approximately 38% in the adult general population and even higher rates among patients with obesity and diabetes, up to 65% [3, 4]. MASLD also carries a risk of progression to steatohepatitis, fibrosis, cirrhosis, and hepatocellular carcinoma [4]. 

Current guidelines emphasize weight loss through lifestyle modification- including diet, exercise, and behavioral counseling- as the cornerstone of MASLD management [5]. Sustained weight reduction of at least 7–10% of body weight has been associated with histologic improvement in steatosis and fibrosis [5, 6]. However, achieving and maintaining this degree of weight loss remains challenging in clinical practice.

In recent years, glucagon-like peptide-1 (GLP-1) receptor agonists, originally developed for type 2 diabetes and obesity, have emerged as promising pharmacologic tools. Randomized trials have demonstrated their efficacy in promoting weight loss, improving insulin sensitivity, and reducing hepatic steatosis [7, 8]. While GLP-1 agonists may offer benefits in MASLD, their comparative effectiveness against physical activity, particularly in terms of liver metrics such as fibrosis scores, controlled attenuation parameter (CAP), and liver stiffness, remains uncertain and an area of ongoing research.

Importantly, MASLD disproportionately affects Hispanic populations, who not only experience higher rates of obesity and type 2 diabetes but also demonstrate more rapid progression to advanced fibrosis compared to non-Hispanic populations [9]. Bexar County, Texas, home to San Antonio, is predominantly Hispanic, and approximately 70% of adults are overweight or obese, exceeding the national average [10]. This high disease burden highlights the urgent need to study management strategies in this predominantly Hispanic population.

In routine practice, lifestyle therapy is variably implemented and rarely standardized or monitored, making it difficult to define retrospectively; physical activity is a first-line recommendation more consistently documented. Therefore, the purpose of this study is to compare the effects of GLP-1 agonists and usual care physical activity without GLP-1 therapy on body mass index (BMI) and non-invasive liver metrics in a disproportionately affected population. We hypothesized that GLP-1 receptor agonist therapy would be associated with greater improvements in BMI and noninvasive liver metrics (CAP, liver stiffness, and FIB-4) than physical activity alone.

## Methods

This retrospective longitudinal chart review analyzed 202 adult patients diagnosed with MASLD and seen from August 1, 2016 to July 31, 2025 at an outpatient gastroenterology research clinic in San Antonio, Texas. MASLD was identified via International Classification of Diseases (ICD)-10 diagnosis codes in the electronic health record, and alternative etiologies were excluded by removing patients with ICD-10 codes for other liver diseases (viral hepatitis, autoimmune/cholestatic liver disease, hereditary liver disease) and for alcohol use disorder or alcohol-associated liver disease. Clinicians diagnosed MASLD based on current MASLD definitions of hepatic steatosis in the setting of at least one cardiometabolic risk factor (overweight/obesity, type 2 diabetes/prediabetes, hypertension, and dyslipidemia).

**Fig. 1:**
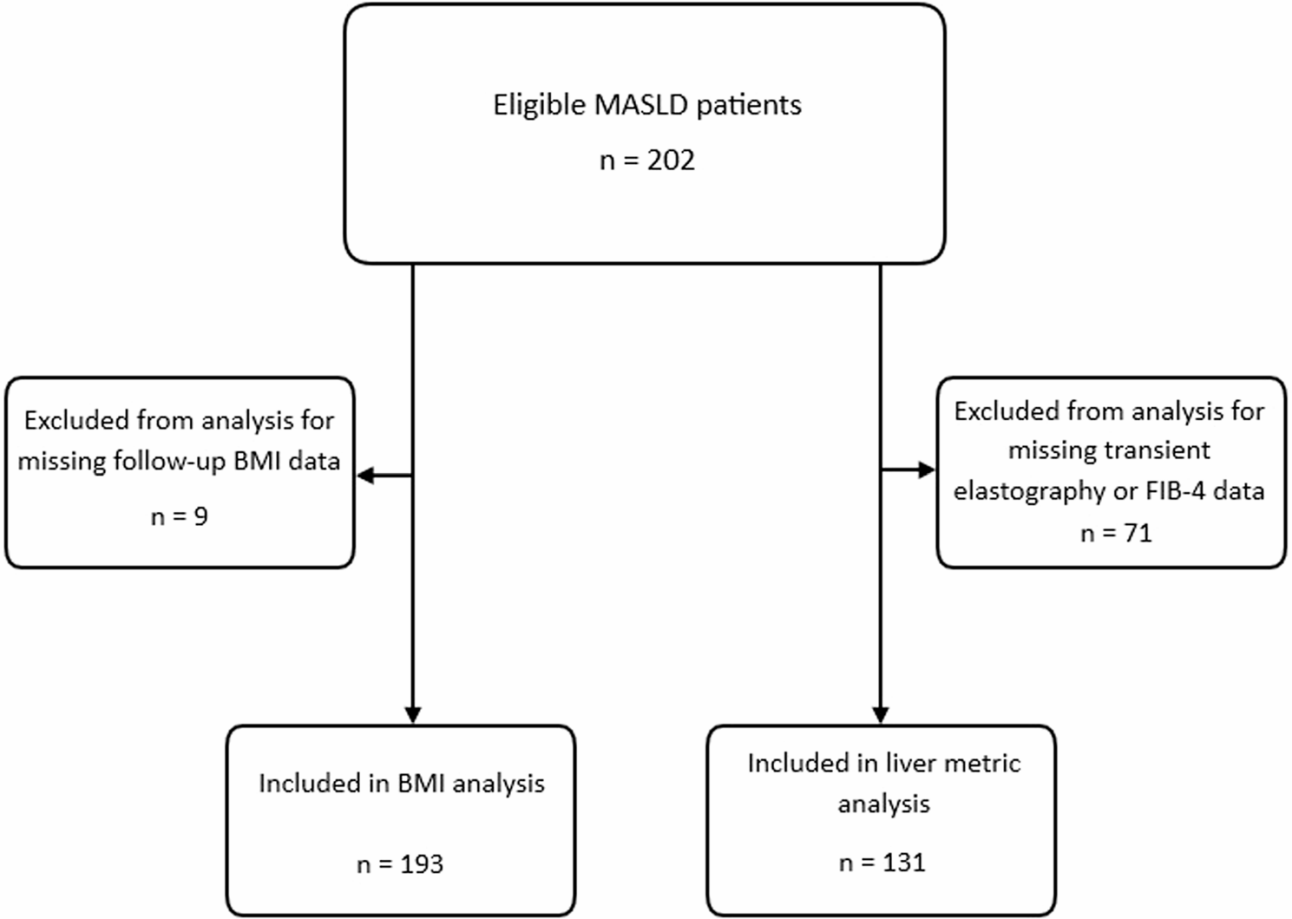
Flow diagram demonstrating inclusion of patients with a complete set of outcomes and exclusion of those missing baseline or follow up data

Data points including demographics, GLP-1 agonist use (tirzepatide or semaglutide for at least 3 months), physical activity, BMI, laboratory values, and fibroscan results were recorded from electronic medical record review at two time points, with a follow-up period ranging from one to two years. This follow-up window was used to reflect variable real-world clinic follow-up and to maximize the number of patients with paired measurements. Patients were included if they had a complete set of data for at least one outcome measure (BMI, laboratory values, or fibroscan) at both time points; those without were excluded (Fig. 1).

Demographic data collected were age at initial presentation, sex, and ethnicity. Physical activity was defined as at least 30 min of cardiovascular exercise three times weekly documented in the electronic health record. Patients were categorized based on GLP-1 receptor agonist use during the observation interval. The GLP-1 group included any patient using a GLP-1 receptor agonist, regardless of reported physical activity. The comparator physical activity group included patients not using GLP-1 therapy who reported meeting the predefined exercise threshold. Thus, group assignment was mutually exclusive. FIB-4 scores were calculated from the age, AST, ALT, and platelet count. Transient elastography examinations were performed as part of routine clinical care by trained operators. Acquisition and quality criteria are described in the Supplementary Methods file. Liver biopsies were not routinely performed in this clinic cohort; therefore, histologic confirmation was not available. This study was determined to be exempt from full review by the Institutional Review Board.

### Data analysis

Baseline demographic and clinical variables were summarized as means with standard deviations for continuous measures and counts with percentages for categorical measures. The primary outcomes were body mass index (BMI), controlled attenuation parameter (CAP), liver stiffness in kilopascals (kPa), and Fibrosis-4 (FIB-4) indices, assessed from baseline to follow-up. Secondary analyses were subgroup analyses by sex and ethnicity. They were conducted as exploratory with conservative interpretation given limited sample sizes within strata.

For each outcome, an analysis of covariance (ANCOVA) was fitted with the post-treatment value as the dependent variable, baseline value as a covariate, and treatment group (GLP-1 agonist vs. physical activity) as the main factor. Modeling post-treatment values with baseline as a covariate, consistent with standard ANCOVA methodology, provides unbiased estimates of treatment effects even when baseline values differ between groups. An expanded multivariable ANCOVA model was fitted for the BMI dataset in which follow-up BMI served as the dependent variable and baseline BMI, age, sex, and ethnicity were included as covariates. This model provided an age-, sex-, ethnicity-, and baseline-adjusted estimate of the comparative effect of GLP-1 therapy on BMI, which was not performed for CAP, liver stiffness, and FIB-4 datasets due to limited sample size. For clinical interpretability, unadjusted change scores (follow-up minus baseline) were also summarized descriptively.

Adjusted mean differences with 95% confidence intervals (CIs) and two-sided p-values were reported for the overall treatment effect. To assess whether treatment effects on BMI varied by subgroup, Type III ANCOVA models including interaction terms were fitted for sex and ethnicity. Stratified ANCOVA models were then used to estimate baseline adjusted treatment differences within each subgroup. This was also performed for CAP and liver stiffness, although due to small sizes in some strata, Type III ANCOVA sex, ethnicity, and treatment interaction tests were not performed. Stratified nor interaction analyses were performed for FIB-4 subgroup analyses because of insufficient subgroup sample sizes. Two-sided p-values < 0.05 were considered statistically significant. A sensitivity analysis was conducted by additionally adjusting for follow-up duration (months) to evaluate whether heterogeneity in visit timing influenced the estimated treatment effects. Model assumptions were assessed by visual inspection of residual Q–Q plots and residuals-versus-fitted plots (Supplementary Fig. 1).

## Results

### Demographics and Baselines

A total of 202 patients with MASLD were analyzed, with 193 of them having a complete set of BMI data at baseline and follow‑up, and 131 having a complete set of liver metric data. The average age was 59.6 years, with 67.8% being female and 32.2% male. 42.6% identified as Hispanic. At the initial visit for MASLD evaluation, the average BMI was 31.5 kg/m² (SD 6.5), and 86% of patients were obese or overweight. Average unadjusted baseline CAP, liver stiffness, and FIB-4 were 290.60 dB/m (SD 54.60), 6.68 kPa (SD 4.13), and 1.24 (SD 0.86), respectively (Table 1). Baseline characteristics comparing participants included versus excluded from the BMI analysis and those with versus without FibroScan data are shown in Tables 2 and 3 respectively. Overall, demographic distributions were similar, whereas patients with FibroScan data tended to be older, had a higher prevalence of diabetes, and higher baseline BMI than those without FibroScan.

**Table 1:** Baseline mean values for BMI, CAP, liver stiffness, and FIB-4 overall and stratified by treatment group (lifestyle modification versus GLP-1 receptor agonist therapy) and by sex and ethnicity

Outcome	Subgroup (*n*)	Overall mean (SD)	Physical activity mean (SD)	GLP-1 mean (SD)
BMI (kg/m²)	Female (132)	30.95 (6.73)	29.80 (5.59)	34.41 (8.55)
	Male (61)	32.69 (5.79)	31.20 (4.17)	36.55 (7.54)
	Hispanic (80)	32.86 (7.59)	30.48 (5.35)	38.10 (9.14)
	Non-Hispanic (113)	30.54 (5.39)	30.07 (5.16)	32.18 (5.97)
	Overall (193)	31.50 (6.48)	30.23 (5.22)	35.14 (8.21)
CAP (dB/m)	Female (58)	285.93 (53.87)	280.68 (53.91)	308.36 (49.85)
	Male (37)	297.92 (55.67)	293.87 (56.06)	315.29 (54.49)
	Hispanic (42)	289.86 (56.99)	281.58 (55.78)	320.22 (53.63)
	Non-Hispanic (53)	291.19 (53.17)	289.00 (54.43)	301.89 (47.89)
	Overall (95)	290.60 (54.60)	285.82 (54.78)	311.06 (50.22)
Stiffness (kPa)	Female (59)	6.18 (3.59)	5.47 (1.84)	9.26 (6.78)
	Male (39)	7.44 (4.78)	6.41 (1.80)	10.88 (8.93)
	Hispanic (44)	7.65 (5.07)	6.02 (1.73)	12.56 (8.11)
	Non-Hispanic (54)	5.89 (2.99)	5.70 (1.97)	6.86 (6.03)
	Overall (98)	6.68 (4.13)	5.83 (1.87)	9.99 (7.64)
FIB-4	Overall (59)	1.24 (0.86)	1.22 (0.83)	1.30 (0.98)

*Abbreviations*: *BMI* body mass index, *CAP* controlled attenuation parameter, *FIB-4* Fibrosis-4 index, *GLP-1 *glucagon-like peptide-1, *kPa* kilopascals, *SD* standard deviation

**Table 2:** Baseline characteristics of patients included versus excluded from the BMI analysis

Characteristic	BMI Included (*n* = 193)	BMI Excluded (*n* = 9)
Age, years	52 ± 11	50 ± 9
Female sex, %	68	67
Hispanic ethnicity, %	43	44
Baseline BMI, kg/m²	34.1 ± 6.2	32.9 ± 7.9
Diabetes, %	54	56
GLP-1 use, %	61	67
CAP, dB/m	318 ± 52	310 ± 48
Liver stiffness, kPa	9.1 ± 4.2	8.7 ± 3.9

Values are reported as mean ± SD. *Abbreviations*: *BMI* body mass index, *GLP-1* glucagon-like peptide-1, *SD* standard deviation, *kPa* kilopascals

**Table 3:** Baseline characteristics of patients included versus excluded from the fibroscan analysis

Characteristic	FibroScan Included (*n* = 131)	FibroScan Excluded (*n* = 71)
Age, years	54 ± 10	47 ± 11
Female sex, %	69	68
Hispanic ethnicity, %	44	41
Baseline BMI, kg/m²	35.2 ± 6.4	31.8 ± 5.9
Diabetes, %	60	38

*Abbreviations*: *ALT* alanine aminotransferase, *AST* aspartate aminotransferase, *BMI* body mass index, *SD* standard deviation, *U/L* units per liter

### BMI

There was an overall unadjusted BMI change of -0.65 kg/m² (SD 3.20; 2.1% decrease) regardless of treatment type. GLP-1 recipients experienced an unadjusted average decrease of 2.04 kg/m² (SD 3.57; 5.8% decrease) compared with 0.16 kg/m² (SD 2.91; 0.5% decrease) in the physical activity group (Table 4). After adjusting for baseline BMI, age, sex, and ethnicity, GLP-1 therapy was associated with a significant reduction in BMI compared with physical activity without GLP-1. Patients receiving GLP-1 agonists had an adjusted post-treatment BMI of 29.77 (95% CI, 28.89–30.66) versus 31.24 (95% CI, 30.73–31.75) in the physical activity group. The adjusted mean difference was − 1.47 kg/m² (95% CI, − 2.54 to − 0.41; *p* = 0.007).

**Table 4:** Unadjusted mean changes from baseline to follow-up and ANCOVA-adjusted between-group differences (GLP-1 vs. lifestyle) for BMI, CAP, liver stiffness, and FIB-4 overall and within sex and ethnicity subgroups

Outcome	Group	Unadjusted mean change (lifestyle)	Unadjusted mean change (GLP-1)	Observed difference (GLP-1 – lifestyle)	Adjusted difference (95% CI)	Test of Significance (ANCOVA), *p*-value
BMI (kg/m²)	Overall	–0.16 (SD 2.91)	–2.04 (SD 3.57)	–1.88	–1.47 (–2.54 to − 0.41)	0.01
	Female	–0.40 (SD 2.48)	–1.80 (SD 2.72)	–1.40	–0.85 (–1.87 to + 0.16)	0.1
	Male	+ 0.37 (SD 3.68)	–2.51 (SD 4.90)	–2.88	–2.54 (–5.10 to + 0.02)	0.05
	Hispanic	+ 0.28 (SD 3.72)	–1.60 (SD 2.45)	–1.88	–1.44 (–3.27 to + 0.40)	0.12
	Non-Hispanic	–0.44 (SD 2.25)	–2.47 (SD 4.44)	–2.04	–1.64 (–2.87 to − 0.41)	0.01
CAP (dB/m)	Overall	–11.66 (SD 55.94)	–28.83 (SD 58.13)	–17.17	–3.31 (–28.79 to + 22.17)	0.8
	Female	–11.53 (SD 62.99)	–52.55 (SD 47.89)	–41.02	–22.55 (–56.51 to + 11.41)	0.19
	Male	–11.87 (SD 43.67)	+ 8.43 (SD 55.69)	+ 20.30	+ 29.10 (–5.78 to + 63.97)	0.1
	Hispanic	–22.21 (SD 58.95)	–31.22 (SD 64.94)	–9.01	+ 16.90 (–21.14 to + 54.94)	0.37
	Non-Hispanic	–3.75 (SD 52.87)	–26.44 (SD 54.32)	–22.69	–16.61 (–51.44 to + 18.21)	0.34
Stiffness (kPa)	Overall	–0.32 (SD 1.75)	–4.29 (SD 8.51)	–3.97	+ 0.01 (–1.04 to + 1.05)	0.99
	Female	–0.29 (SD 1.48)	–2.67 (SD 7.55)	–2.38	+ 0.96 (–0.41 to + 2.33)	0.17
	Male	–0.37 (SD 2.13)	–6.28 (SD 9.62)	–5.91	–1.36 (–2.86 to + 0.13)	0.07
	Hispanic	–0.40 (SD 2.01)	–5.94 (SD 9.92)	–5.54	+ 1.71 (–0.11 to + 3.53)	0.07
	Non-Hispanic	–0.26 (SD 1.55)	–2.28 (SD 6.37)	–2.02	–1.07 (–2.20 to + 0.07)	0.06
FIB-4	Overall	–0.02 (SD 0.48)	–0.35 (SD 0.82)	–0.33	–0.29 (–0.56 to − 0.03)	0.03

*Abbreviations*: *ANCOVA* analysis of covariance,* BMI* body mass index, *CAP* controlled attenuation parameter, *CI* confidence interval, *FIB-4* Fibrosis-4 index, *GLP-1* glucagon-like peptide-1, *kPa* kilopascals, *SD* standard deviation

In exploratory stratified analyses, GLP-1 therapy in females was associated with an adjusted mean BMI reduction of 0.85 kg/m² compared with physical activity without GLP-1 (95% CI, − 1.87 to + 0.16; *p* = 0.10), while males had a larger adjusted reduction of 2.54 kg/m² (95% CI, − 5.10 to + 0.02; *p* = 0.052). Among non-Hispanic participants, GLP-1 use was associated with a significant adjusted mean reduction of 1.64 kg/m² (95% CI, − 2.87 to − 0.41; *p* = 0.009), whereas in Hispanic participants the reduction was 1.44 kg/m² (95% CI, − 3.27 to + 0.40; *p* = 0.123).

However, formal interaction testing in the full Type III ANCOVA model found no statistically significant effect modification (treatment×sex *p* = 0.37; treatment×ethnicity *p* = 0.39). While point estimates differed across strata, these exploratory subgroup contrasts did not provide statistically robust evidence of differential treatment response by sex or ethnicity.

### CAP

There was an overall unadjusted CAP change of -14.91 dB/m (SD 56.45; 5.1% decrease) regardless of treatment type. Patients receiving GLP‑1 agonists exhibited a larger unadjusted mean CAP reduction of 28.83 dB/m (SD 58.13) versus 11.66 dB/m (SD 55.94) in the physical activity group. After adjusting for baseline CAP, GLP‑1 therapy did not demonstrate a significant overall effect on liver steatosis compared with physical activity alone, but directionally, the average CAP was reduced by 3.31 dB/m more among GLP-1 users (95% CI, − 28.79 to 22.17 dB/m; *p* = 0.80).

In exploratory stratified ANCOVA analyses, CAP point estimates differed by sex and ethnicity, but none of the subgroup contrasts reached statistical significance. In females, GLP-1 therapy was associated with an adjusted mean CAP change of − 22.55 dB/m versus physical activity alone (95% CI, − 56.51 to 11.41; *p* = 0.19). In males, GLP-1 therapy was associated with an adjusted mean CAP change of + 29.10 dB/m relative to physical activity (95% CI, − 5.78 to 63.97; *p* = 0.10). Among non-Hispanic participants, the adjusted difference was − 16.61 dB/m (95% CI, − 51.44 to 18.21; *p* = 0.34), whereas among Hispanic participants it was + 16.90 dB/m (95% CI, − 21.14 to 54.95; *p* = 0.37).

### Liver Stiffness

There was an overall unadjusted liver stiffness change of -1.13 kPa (SD 4.38; 16.9% decrease) regardless of treatment type. GLP-1 recipients experienced a greater mean unadjusted reduction of 4.29 kPa (SD 8.51) compared with a 0.32 kPa (SD 1.75) reduction in the physical activity group. After adjusting for baseline liver stiffness, however, GLP-1 therapy was not associated with a significant difference in stiffness compared with physical activity without GLP-1 use (adjusted mean difference + 0.01 kPa; 95% CI, − 1.04 to + 1.05; *p* = 0.99).

In exploratory stratified analyses, none of the subgroup contrasts reached statistical significance. Females receiving GLP-1 had a mean liver stiffness change 0.96 kPa higher than the physical activity group (95% CI, − 0.41 to 2.33; *p* = 0.17), whereas males receiving GLP-1 had a mean stiffness 1.36 kPa lower (95% CI, − 2.86 to 0.13; *p* = 0.07). Among non-Hispanic participants, the adjusted difference was − 1.07 kPa (95% CI, − 2.20 to 0.07; *p* = 0.06), while among Hispanic participants it was + 1.71 kPa (95% CI, − 0.11 to 3.53; *p* = 0.07).

### FIB-4

Regarding FIB-4 indices, there was an overall unadjusted change of -0.12 (SD 0.61; 9.5% decrease) regardless of treatment type. Those on GLP-1 therapy had a greater mean reduction of 0.35 (SD 0.82) compared to a 0.02 (SD 0.48) reduction in the physical activity group. Furthermore, even after adjusting for baseline indices, the GLP-1 group had a statistically greater reduction in FIB-4 than physical activity without GLP-1 therapy (adjusted mean difference − 0.29; 95% CI − 0.56 to − 0.03; *p* = 0.029). However, this was not clinically significant, as none of the patients in either group improved from higher risk (> 2.67) to low risk (< 1.3) indices.

In sensitivity analyses adjusting for follow-up duration (months), the adjusted differences between the GLP-1 and physical activity groups did not meaningfully change in direction or magnitude across any primary outcome.

## Discussion

Overall, both GLP-1 agonist use and physical activity without GLP-1 therapy were associated with improvements in BMI and noninvasive liver metrics (FIB-4, CAP, stiffness). Prior to adjusting for baseline differences, GLP-1 agonist users had greater improvements in several key outcomes compared to physical activity without GLP-1 use: mean BMI decreased by 5.3% more in the GLP-1 group, CAP declined by 5.2% more with GLP-1 agonists, liver stiffness (kPa) decreased by 37.5% more with GLP-1 agonists, and FIB-4 fell by 25.3% more with GLP-1 agonists. However, patients receiving GLP-1 therapy had higher baseline values on average, which may have biased results, as higher starting levels are often associated with greater potential for change. Therefore, after adjusting for baseline differences between the two treatment groups, GLP-1 users had statistically significantly greater reductions only in BMI and FIB-4 indices. However, the clinical significance of FIB-4 reduction is ambiguous as none of the patients in either group improved from higher risk (> 2.67) to low risk (< 1.3) indices.

The pattern of results fits within the context of current literature showing that GLP-1 receptor agonists produce meaningful weight loss and favorable hepatic effects beyond simple weight reduction [11–13]. Semaglutide and other GLP-1 agents have demonstrated reductions in liver steatosis [14] and, in some trials, histologic resolution of steatohepatitis and improvements in fibrosis endpoints [8]. Further, GLP-1 agonists have been associated with improved surrogate fibrosis indices like FIB-4, AST-to-platelet count ratio index (APRI), non-alcoholic fatty liver disease fibrosis score (NFS), and liver stiffness [15–17]. Likewise, studies have also shown reductions in lipotoxicity and hepatic inflammation with GLP-1 use [7]. This supports the physiologic mechanism of the larger decreases in FIB-4 and kPa seen here among GLP-1 users, since GLP-1 effects include improved insulin sensitivity, reduced de-novo lipogenesis, and anti-inflammatory actions that could affect fibrosis surrogates [7, 8]. 

By contrast, guideline-based lifestyle intervention, while established as the cornerstone of MASLD management and shown to produce histologic improvement when 7–10% weight loss is achieved, often proves difficult to implement and sustain in clinical practice [6]. The modest BMI and liver-metric changes observed in the physical activity group of this study may be reflective of adherence challenges and thus more limited practical effectiveness. Because lifestyle counseling is routine, some patients receiving GLP-1 therapy may have also exercised in this real-world study. This supports an approach in which pharmacotherapy can be used to supplement lifestyle interventions to help patients achieve clinically meaningful weight loss and liver benefit when lifestyle change alone is insufficient or not durable, especially in settings when comorbidities may limit physical activity or dieting ability.

The cohort’s exploratory demographic findings underscore important equity considerations. Descriptively, there was a greater BMI reduction difference between treatment groups for males (–2.54 kg/m²) compared to females (–0.85 kg/m²). Interestingly, males had an increase in average absolute BMI on follow up with physical activity in the absence of GLP-1 therapy, which may have contributed to the greater difference between treatment groups. Females, on the other hand, lost almost half a kg/m^2^ on a physical activity regimen only, which may explain why they did not experience as much of a benefit from GLP-1 agonists on average than males did. This is contrasted to current literature that females experience greater weight loss compared to males on GLP-1 agonist receptors [18, 19]. The difference between treatment groups did not meaningfully differ between Hispanics and non-Hispanics. No consistent patterns were observed when comparing treatment methods within sex and ethnicity subgroups for the non-invasive liver metrics. Nevertheless, these real-world, exploratory findings may provide a useful benchmark for future studies examining equity considerations in MASLD treatment.

## Limitations

This study is limited by its sample size and exploratory design, which constrained the ability to draw concrete conclusions about demographic differences; therefore, subgroup analyses comparing sex and ethnicity, especially for liver metrics with smaller samples, were exploratory. Requiring paired measurements may preferentially include more engaged patients with complete follow-up, introducing selection bias. Physical activity behaviors were self-reported and difficult to standardize, with variable adherence, which may contribute to measurement bias. In clinical practice, compliance to lifestyle interventions cannot be enforced. Baseline metabolic comorbidities beyond BMI and liver metrics (diabetes, dyslipidemia, hypertension) were also not fully adjusted for, raising the possibility of residual confounding.

The follow-up window of one to two years reflects real-world visit variability but introduces heterogeneity in exposure duration that may influence outcomes. However, sensitivity analyses adjusting for follow-up duration showed similar direction and magnitude of effects across all primary outcomes. This duration may still be insufficient to detect meaningful fibrosis regression. Dose, titration schedules, and adherence could not be reliably ascertained from the retrospective record review for all patients; therefore, analyses treated GLP-1 exposure as a class-level indicator rather than evaluating dose-response or persistence.

However, the strength of this study is its real-world clinical setting, which captures the complexity of MASLD management beyond the constraints of controlled trials. The inclusion of diverse, routine clinic patients- many with comorbidities and variable follow-up intervals- enhances the generalizability of the findings to everyday practice. By accounting for factors such as inconsistent adherence, heterogeneous physical activity engagement, and real-world prescribing patterns, this analysis reflects the therapeutic effectiveness of GLP-1 agonists and physical activity as they occur in practice, rather than idealized efficacy under trial conditions. This pragmatic design allows a more realistic appraisal of how these interventions perform among the populations most affected by MASLD.

## Conclusion

In this real-world cohort of patients with MASLD, GLP-1 receptor agonist therapy was associated with greater improvements in BMI and FIB-4 compared to physical activity without GLP-1 therapy, while baseline-adjusted differences in CAP and liver stiffness were not statistically significant. The consistent directionality of benefit across metabolic and hepatic measures may support the potential complementary role of GLP-1 agents in MASLD management. Demographic subgroup exploratory analyses revealed no clear demographic trends in treatment response across sex or ethnicity. Given the challenges of adherence and sustained weight loss in routine practice, these real-world findings complement randomized trial evidence and support the importance of combining pharmacologic and lifestyle strategies to achieve meaningful outcomes, particularly in Hispanic and obese populations with high disease prevalence. Future prospective studies with larger, ethnically representative samples and standardized follow-up are warranted to clarify subgroup effects and confirm long-term hepatic benefits.

## Supplementary Information

Supplementary Material 1.

Supplementary Material 2.

## Data Availability

The dataset collected and analyzed for the study is available from the corresponding author on reasonable request.
