# Supplementary material for: Real-world comparison of GLP-1 agonists versus physical activity in metabolic dysfunction-associated steatotic liver disease
Source: BMC Gastroenterol. 2026 Feb 25;26:198. doi: 10.1186/s12876-026-04626-7 (PMC13040818; doi:10.1186/s12876-026-04626-7)
Supplement: Supplementary file 1 — Supplementary Material 1. [file 12876_2026_4626_MOESM1_ESM.docx]

Supplemental Figure 1. Model diagnostic plots (Q–Q and residuals versus fitted) for BMI, CAP, liver stiffness measurement (kPa), and FIB-4 models.
