# Supplementary material for: Real-world comparison of GLP-1 agonists versus physical activity in metabolic dysfunction-associated steatotic liver disease
Source: BMC Gastroenterol. 2026 Feb 25;26:198. doi: 10.1186/s12876-026-04626-7 (PMC13040818; doi:10.1186/s12876-026-04626-7)
Supplement: Supplementary file 2 — Supplementary Material 2. [file 12876_2026_4626_MOESM2_ESM.docx]

Supplementary Methods. FibroScan methodology and details on probe selection, fasting state, reliability criteria (IQR/median), and operator technique.

Transient elastography (FibroScan®, Echosens, Paris, France) was performed to assess liver stiffness (kPa) and steatosis using controlled attenuation parameter (CAP, dB/m). Examinations were performed by trained operators following manufacturer and institutional protocols, with patients fasting. Patients were placed in a supine position for 5 minutes prior to the start of the exam. Using the M probe or XL probe, which was selected according to body habitus per operator assessment, the center of the liver parenchyma was located using TM and A-mode ultrasound. Once positioned, the probe induced a 50 Hertz shear wave, and a series of no less than 10 ultrasound transient elastography measurements were performed. Each measurement produced a propagation map image which was analyzed to confirm parallel shear wave margins. These images were analyzed to determine shear wave propagation speed and calculate the equivalent liver stiffness. All images were reviewed by the operator and the physician for technical accuracy. Studies were included if they met standard reliability criteria (IQR/median for liver stiffness ≤0.30). The median values for liver stiffness and CAP were recorded.
